# The Interpersonal Theory of Suicide and Relationship Satisfaction: A Daily Diary Study

**DOI:** 10.3390/bs14121138

**Published:** 2024-11-27

**Authors:** Heather A. Love, Preston Morgan

**Affiliations:** 1 Department of Human Development and Family Studies, University of Alabama, Tuscaloosa, AL 35487, USA; 2 Department of Human Development and Family Science, Oklahoma State University, Stillwater, OK 74078, USA; preston.morgan11@okstate.edu

**Keywords:** couples, Interpersonal Theory of Suicide, romantic relationships, suicide ideation

## Abstract

Romantic relationships serve as one of the most important relationships in adults’ lives, yet the influence of relational dynamics on suicide ideation (SI) is limited and longitudinal associations are unclear. The Interpersonal Theory of Suicide has been applied to romantic relationships broadly and supports motivations of suicide (thwarted belonging and perceived burdensomeness) and relationship satisfaction to be predictors of SI. An online daily diary study (*n* = 94 adults) was conducted to examine romantic relationship dynamics and mental health indicators in adults over 10 days. Multilevel growth modeling results revealed that higher perceived burdensomeness, but not relational satisfaction or thwarted belonging, was associated with higher initial levels of SI. However, perceived burdensomeness, thwarted belonging, and relationship satisfaction were not associated with rates of change in SI over time. Further, no interaction effects between either relationship satisfaction and thwarted belonging or perceived burdensomeness were found in association with trajectories of SI over the 10-day period. The results of this study indicate that relational satisfaction, through a commonly used global assessment of relational wellbeing, may not have a substantial influence on SI, particularly in individuals with mild SI levels. This may be due to relational satisfaction being relatively consistent, while SI is prone to short-term fluctuations. However, additional research is recommended to address other relational dynamics’ influences on SI.

## 1. Introduction

Despite significant prevention efforts, suicide continues to present an expanding crisis in the United States [1]. While the predominant focus on suicide prevention and intervention is limited to the individual alone [2], prominent theories of suicide emphasize the significance of connection and integration in interpersonal relationships [3,4,5,6]. Romantic relationships serve as one of the most important interpersonal relationships in adult life, providing both connection and integration with others. Previously, simply participating in a committed romantic relationship was perceived as a protective factor against suicide (see [7] for review); however, more recent research has determined that the quality, not merely the presence, of the relationship needs to be explicitly assessed to determine both protective and risk factors associated with romantic relationships and suicidality [8].

A primary indicator of the quality of romantic relationships is relational satisfaction. Relationship satisfaction broadly represents the degree to which individuals are happy with their relationship and is therefore one of the most prominently studied relationship dynamic variables. Assessing satisfaction in relationships also provides valuable information about other meaningful processes, as satisfaction is associated with a range of other relational dynamics, including relational stability [9,10], commitment to the relationship [11], and dyadic coping [12]. Finally, higher relational satisfaction is associated with both physical and psychological health [13,14,15] as well as overall life satisfaction [10]. Based on the significance of relationship satisfaction in both individuals and couples, understanding its role in the development, sustainment, or declines in suicidal ideation and behaviors is paramount. For clinicians and researchers alike, there is a significant need to better understand the acute and long-term influences of romantic relationships to better prevent and intervene with suicide ideation (SI).

A significant body of research reveals the substantial association between relationship satisfaction (and its inverse, relational distress) and suicide ideation and behaviors, in which relational distress is a risk for suicide ideation and behaviors and satisfaction is identified as a protective factor against ideation and behaviors [2,16,17,18,19,20,21,22,23]. However, nearly all research documenting this association to date is cross-sectional. Two prominent challenges arise when attempting to draw conclusions about SI from cross-sectional research: (1) retrospective accounts about SI are frequently vague or exaggerated [24,25]; (2) causal processes cannot be detected. There are only a few longitudinal studies that have examined how relationships influence suicidality (particularly relationship separation and dissolution [26,27,28]), but only one longitudinal study to date has been conducted that revealed low relationship satisfaction to be predictive of suicide risk six months later [16]. This study advances the evidence that relational satisfaction is not only associated with, but predictive of, suicidality using a longitudinal survey design.

Despite the emerging evidence of a causal association between relational satisfaction and SI, additional research is necessary to better understand these impacts to provide guidance for prevention and intervention efforts. For instance, recent studies using intensive longitudinal methods, in which participants are surveyed frequently over a set period [29], have determined that SI fluctuates frequently and sometimes dramatically over the course of days or even hours [30,31]. The causes of these fluctuations, however, are not clearly identified [32]. Given the substantial evidence of relational satisfaction’s association with SI, understanding how relational satisfaction influences both immediate SI as well as changes in SI can provide opportunities for intervention or prevention. This study seeks to fill these gaps using a prominent theory of suicide in a daily dairy study.

## 2. Interpersonal Theory of Suicide

The Interpersonal Theory of Suicide (ITS) [5,33] is one of the leading theories of suicide to date. The ITS is useful in understanding both motivations for suicide as well as the potential risk for suicide from a systemic framework. The ITS incorporates two primary experiences that theoretically serve as motivation for suicide: thwarted belonging and perceived burdensomeness. Thwarted belongingness refers to experiences of loneliness and isolation, while perceived burdensomeness is indicated when the individual considers themselves a burden on others or has the belief that others would be better off without them. Finally, the ITS summarizes how an individual may be able to end one’s own life via acquired capability [33].

Despite this theory’s prominent attention to the role of interpersonal connectedness, this theory has not been directly tested in romantic relationship research [17]. The robust literature supporting the associations between perceived burdensomeness and thwarted belonging and SI [34,35] have yet to incorporate the additional impact of relational satisfaction, which may significantly alter how these motivations affect SI. In other words, while relationship satisfaction and SI have a known association, this association has the potential to be amplified when perceived burdensomeness or thwarted belonging is also present. For instance, individuals who experience high thwarted belonging may experience higher rates of SI when relational satisfaction is low due to the compounded impact of feeling disconnected or lonely in their relationships and more generally. Similarly, a person who feels others would be better off if they were dead (i.e., perceived burdensomeness) may be particularly affected if their relationship satisfaction is low, leading to a stronger association with SI when both are present. Thus, this study sought to incorporate motivations for suicide, thwarted belonging and perceived burdensomeness, to assess whether these moderate the association between relationship satisfaction and SI.

Although the theoretical foundation has been set for romantic relationships to be examined in motivations for suicide, the specific roles of thwarted belonging and perceived burdensomeness have yet to be assessed. The present study seeks to address this gap in the literature by examining perceived burdensomeness and thwarted belonging as predictors of SI over time as well as moderators to the association between relationship satisfaction and changes in SI over time.

## 3. Present Study

To investigate how relationship satisfaction was associated with changes in SI over a 10-day period, we conducted a daily diary study [36] about SI and relationship dynamics each day. This study builds on a prior study [37], in which rates of SI were found to decline over a 10-day period. The purpose of this study was to investigate the associations between romantic relationship satisfaction and SI. Specifically, we theorized that motivations for suicide (i.e., thwarted belonging and perceived burdensomeness) would explain the context for when relational satisfaction would associate with changes in SI over a 10-day period. Without this theoretical application and robust longitudinal examination, clinicians and researchers alike are limited in understanding how relationships specifically impact SI over time. To address these gaps in the literature, we sought to answer the following research questions:

RQ1: Does relationship satisfaction predict initial levels of suicidal ideation and rates of changes in suicidal ideation trajectories over time?

RQ2: Does perceived burdensomeness and thwarted belonging moderate the association between relationship satisfaction and suicidal ideation trajectories over time?

## 4. Method

### 4.1. Participants and Procedures

All participants were recruited from Prolific, an online research recruitment platform. Participants who met basic inclusion criteria (lived in the United States, at least 19 years of age, in a romantic relationship, and experienced depressive symptoms) based on their pre-screener responses on Prolific’s website were eligible to view this study. Those who met these criteria completed a baseline assessment, which also included consent to participate in this study. The baseline survey then confirmed each of the inclusion criteria and assessed for the presence of SI; only participants who indicated SI (as assessed by the Suicidal Ideation Attributes Scale, described below) were invited to participate in the daily diary study. In total, 200 participants completed the baseline assessment, 103 of whom indicated SI; of these, 94 participants elected to continue participating in the daily diary study.

The daily diary study began two days after the baseline assessment. After consenting to participate in the daily diary study (included in Day 1), participants were able to complete one survey per day for 10 days during the daily diary period. Each daily survey was available for 24 h; at midnight, the next day’s survey was released. Daily surveys took five minutes or less to complete. Each daily diary survey included measures adapted for daily surveys to assess mental health (SI and motivations for suicide) and romantic relationship dynamics (e.g., satisfaction, communication, and conflict) each day. Participants received immediate compensation for each daily survey (USD 1) and a bonus payment (USD 2) if all 10 daily diary surveys were completed. This study was approved by the first author’s Institutional Review Board.

### 4.2. Sample Characteristics

This sample of adults at baseline was fairly evenly split: women (47.90%), men (49.90%), preferred not to answer (1.10%), and other (1.10%). Most participants identified as White (76.59%), with some identifying as Alaskan Native or Native American (1.06%), Asian (6.389%), Black or African American (9.57%), Hispanic or Latino (12.76%), Native Hawaiian or Pacific Islander (1.06%), and Other (1.06%); of these, 6.38% identified as more than one race. Many adults had a 4-year college degree or higher (45.70%) and some were unemployed (26.60%). On average, these adults were 37.47 years old (Median = 34.50, SD = 12.46, Range = 20–77) and had been in current romantic relationship for 9.21 years (SD = 9.21, Range = 0–55). Many adults were married (40.40%) or seriously dating (44.70%), with some casually dating (1.10%), engaged (9.60%), or something else (4.30%). Half of adults did not have any children (54.30%), with some having one child (24.50%), two children (9.60%), three children (9.60%), or more (2.10%). In terms of mental health, some were currently in mental health treatment (38.30%). On average, these adults had experienced some level of suicidal ideation for 0.59 years (SD = 10.15, Range = 0–37).

## 5. Measures

### 5.1. Suicide Ideation

The five-item Suicidal Ideation Attributes Scale (SIDAS) [38] was used to assess suicidal ideation, which evaluates frequency of and control over SI, likelihood of attempting suicide, the extent to which the person is tormented by the SI, and how much SI interferes with daily functioning. Given the daily diary design, the SIDAS was modified to reflect SI that day during the daily diary surveys. All items were rated from 0 (lowest) to 10 (highest). At baseline, participants were given all five items. To be compatible with the daily diary design (particularly burden to participants), if participants indicated a 0 on the first item (no SI), their score was a 0 for the entire scale and they were not given the remaining four items [38]. The second item was reverse-coded [38]. Then, all items were summed together so that higher scores indicated more significant SI. The SIDAS had high internal reliability at baseline (*α* = 0.81).

### 5.2. Motivations for Suicide

Perceived burdensomeness and thwarted belonging were assessed at baseline by the Interpersonal Needs Questionnaire [39]. All items were rated from 1 (Not at all true for me) to 7 (Very true for me), with five reverse-coded items. Six items measured perceived burdensomeness, which included items “These days, the people in my life would be better off if I were gone” and “These days, the people in my life would be happier without me”. These six items were summed together so that higher scores indicated higher perceived burdensomeness (Range 6–42), which had a Cronbach’s alpha of 0.95. Nine items measured thwarted belonging, which included items “These days, I feel like I belong” and “These days, I feel disconnected from other people”. These nine items were summed together so that higher scores indicated higher thwarted belonging (Range 7–63), which had a Cronbach’s alpha of 0.89.

### 5.3. Relationship Satisfaction

The relationship satisfaction subscale (three items) from the Perceived Relationship Quality Component Scale (PRQC) [40] assessed participant level of satisfaction with their romantic relationship at baseline. These items (e.g., “How happy are you with your relationship?”) were rated from 1 (Not at all) to 7 (Extremely), which were then averaged together so that higher scores indicated higher levels of relationship satisfaction. This subscale had a Cronbach’s alpha of 0.97, consistent with the previously established validity (0.93 [40]).

### 5.4. Covariates

We used two covariate variables for the analyses, including age (continuous) and gender (Woman = 1, Man and Non-Binary = −1).

## 6. Analytic Plan

We conducted the analytic plan in several steps, which were all conducted in the SPSS. First, we conducted preliminary analyses, such as Pearson bivariate correlations, to examine the associations between the key predictors and SI at all time points. Descriptives for included variables are found in Table 1. Next, we identified multivariate outliers among participants (i.e., level 2) by the Mahalanobis Distance, which estimates how far a participant is from the mean of the multivariate distribution, taking into account correlations among variables [41]. This revealed no outliers. Second, we used multilevel growth modeling to test the research questions. Specifically, participants’ scores at each day (level 1) were nested within each participant (level 2). Multilevel modeling (MLM) is recommended for small samples compared to structural equation modeling [42]. Furthermore, we followed recommendations for MLM with small samples by using an iterative process to build the model, limit the number of random effects, and handle missing data with restricted maximum likelihood (REML) as well as Kenward–Roger degrees of freedom correction [43,44]. We began with an unconditional multilevel growth model with the initial levels (e.g., intercept) of SI and linear rates of changes (i.e., slope) across the daily dairy study as fixed and random effects. Although conceptually SI may fluctuate, there were very small fluctuations in SI across the 10 days. Given this and the small sample size, we estimated linear rates of change for a parsimonious model. Because we include the baseline assessment, which started two days prior to the 10-day daily diary study, we coded the linear rates of changes in days (i.e., 0, 2, 3, 4, 5, 6, 7, 8, 9, 10, and 11). Then, in following common multilevel procedures [45], linear rates of changes as well as all continuous predictors and covariates were grand mean centered. Next, we ran conditional multilevel growth models through an iterative process that included the key predictors (RQ1), then the interaction terms (relationship satisfaction x perceived burdensomeness; relationship satisfaction x thwarted belonging) (RQ2), and then covariates for the full model. Deviance tests (i.e., chi-square difference tests) evaluated the model fit, where lower log-likelihood values indicated a more parsimonious model.

### Missingness

Missingness ranged from 0% as the lowest amount of missing (all variables at baseline) to 33% as the highest amount of missing (i.e., SI at day 10). A prior study using these data evaluated the changes in SI in the daily diary study [37] and found that 50% of participants completed all 10 days, with most participants completing an average of 7.58 days. Sensitivity analyses in that study revealed that the rates of change in SI did not differ between those who completed all 10 days and those who did not complete all 10 days. Furthermore, we evaluated missingness assumptions for this study by conducting Pearson bivariate correlations of missingness on SI at each time point (i.e., missingness = 1, observed = 0) with the predictors and covariates. Generally, these results provided some evidence of missingness at random (i.e., variables in the model accounted for missingness in the model). For example, identifying as a woman was associated with more missingness on SI at Day 1 (*r* = 0.21) and Day 6 (*r* = 0.26), while identifying as straight or heterosexual was associated with less missingness on SI at Day 1 (*r* = 0.20), Day 6 (*r* = 0.20), Day 8 (*r* = 0.24), and Day 10 (*r* = 0.29). Based on this, we used REML and Kenward–Roger degrees of freedom corrections to handle missing data [44].

## 7. Results

Pearson bivariate correlations reveal that relationship satisfaction at baseline was not associated with SI at any time point, including baseline. These correlations provide evidence that relationship satisfaction may not have an association with SI in this sample. Higher perceived burdensomeness and thwarted belonging were both associated with higher SI at baseline and other days throughout the 10-day study period (see Table 2 for details). Furthermore, higher relationship satisfaction was not associated with perceived burdensomeness (*r* = −0.06, *p* = 0.545), but it was associated with lower thwarted belonging at baseline (*r* = −0.45, *p* < 0.001).

## 8. Multilevel Growth Modeling

The unconditional multilevel growth model yielded convergence issues, which were likely due to the random effects. Specifically, the variance for the rates of changes in SI was 0 and the covariance between the rates of changes with the initial levels was small (*b* = −0.39, *p* = 0.173). The convergence issues were resolved when we removed the random effects for the rates of changes in SI. The results revealed that the initial levels varied across adults (σ^2^ = 20.30, *p* < 0.001) and that SI decreased by –0.47 each day in the daily diary study (*p* < 0.001).

Next, we added the key predictors to develop the conditional multilevel growth model (RQ1), which improved the model fit and was a more parsimonious model (χ^2^ (6) = 24.98, *p* < 0.001). We summarize the results here with *p* < 0.05 (see Table 3 for more details). This revealed that perceived burdensomeness was the only key predictor that was associated with SI at baseline. Specifically, higher perceived burdensomeness at baseline was associated with higher SI at baseline (*b* = 0.21).

Next, we added the interaction terms to the conditional model (RQ2), which did not improve the model fit (χ^2^ (4) = 2.66, *p* = 0.61). The interactions of relationship satisfaction x perceived burdensomeness and relationship satisfaction x thwarted belonging were not associated with the initial levels of SI nor the rates of changes in SI over the daily dairy study. The results from the conditional model remained in this interaction model. Finally, we added the covariates (age and gender) to the conditional model, which did not improve model fit (χ^2^ (4) = 1.78, *p* = 0.76). Neither age nor gender was associated with the initial levels of SI or the rates of changes in SI. However, like the previous models, higher perceived burdensomeness remained a predictor of initial levels of SI.

## 9. Discussion

This study provided important findings regarding the association between relationship satisfaction and SI, with consideration of how motivations for suicide impact these associations. Although the participants in the survey had a notable SI risk at baseline, this risk had a linear decrease over the 10 days of the daily diary. However, these averages remained above the SI cut-off of 1 throughout the daily diary study. This finding supports the recommended practice of frequently assessing suicidal ideation and risk in clinical work.

Surprisingly, relational satisfaction was not associated with either initial levels of SI or changes in SI over the 10 days (RQ1). While prior evidence establishes an association between relational satisfaction and SI, this association was not identified in the current study. There are three possible explanations for this unexpected finding. First, it is possible that this sample had self-selection bias, in which participants with higher-than-average rates of relationship satisfaction (*M* = 5.2 out of a possible 7) were more likely to participate. This finding may reveal that high relational satisfaction was not impacting the SI at baseline or during the daily diaries; therefore, it may be the case that low relationship satisfaction is a more prominent risk factor for SI than high relationship satisfaction is a protective factor against SI [22,46]. In prior studies, couples with high levels of relationship satisfaction were also found to have lower rates of SI [22], which was shown in our sample. It is common for suicide measures to be conservative in determining risk: for example, a score of 1 meets a cut-off for suicidal ideation on the measure used in this study (range 0–50). This conservative assessment method is employed because any risk of someone ending their life is serious. However, the levels of SI in our sample decreased after baseline. One reason that an association between relationship satisfaction and changes in SI was not identified may be due to this sample being more satisfied than average with their relationship, along with having lower SI over time. The brief period of participant experiences may have also led to this lack of finding, in which participants may have been unlikely to experience acute levels of crisis during the 10-day period.

Future research is encouraged to evaluate thresholds of relational satisfaction in conjunction with suicide risk to offer a clearer understanding of this association. Currently, the satisfaction subscale of the Perceived Relationship Quality Component Scale does not include a standardized cut-off for relationship distress. Given that low relationship satisfaction may be important for future research, we encourage the use of relationship satisfaction measures that include cut-offs for relationship distress, such as the Revised Dyadic Adjustment Scale [47] or the Couple Satisfaction Index [48]. Including a relationship measure with established cut-offs may offer a clearer understanding of this association.

A second explanation for these findings includes how relationship satisfaction is commonly identified as a relatively static measure of relational wellness. Similar to the first explanation, if the relationship is relatively consistent it may be the case that a consistently happy relationship has little bearing on SI over a brief period of time, such as this 10-day diary study. This may be explained by the association found by Blow and colleagues [16], in which lower relationship satisfaction was associated with SI six months later. Future research may address this by increasing the length of time between assessments to investigate changes over broader periods.

A final possibility for this lack of significance may also be due to the sample size, which may have reduced statistical power. Thus, it is possible that these associations were non-significant merely due to a lack of statistical power, obfuscating associations that may have been present. Hence, we encourage future studies to replicate these analyses using larger samples.

Of the motivations for suicide, thwarted belonging and perceived burdensomeness, only perceived burdensomeness had a significant association with SI at baseline. However, perceived burdensomeness did not predict changes in SI over the 10 days, nor did it moderate the association between relational satisfaction and SI. While keeping in mind the potential reasons for the lack of associations explained above, particularly related to power, this lack of moderation provides a new theoretical understanding for the association between relationship satisfaction and changes in SI. For example, not only was relationship satisfaction not associated with SI, but it was not better explained by the context of perceived burdensomeness.

This could mean that even when partners feel like a burden to themselves and those around them, it does not better explain when relationship satisfaction is associated with changes in SI. Similar to prior work that theorized that relational satisfaction is associated with thwarted belonging [17], it is perhaps not surprising that when relational satisfaction was not significantly associated with SI in this sample, neither was thwarted belonging. In other words, not only was relationship satisfaction not associated with SI, but it was not better explained by the context of thwarted belongingness. For instance, even when partners feel alone or isolated from those close to them, it does not better explain when relationship satisfaction is associated with changes in SI. The lack of association possibly questions whether the ITS applies to romantic relationships to the extent that prior scholarship has indicated (see [17]); however, more evidence is needed to investigate these associations.

Perceived burdensomeness, on the other hand, was significantly associated with initial SI, but did not reveal any interaction effects when relationship satisfaction was added. This finding is aligned with prior research that has established that perceived burdensomeness is likely a stronger influence on SI than thwarted belonging [34,35]. However, the lack of interaction effect reveals that the feeling of being a burden or that others would be better off if the individual were dead is associated with worsened or alleviated SI in the context of relationship satisfaction. It may be the case that while these interpersonal motivations for suicide (particularly perceived burdensomeness) are connected to perceptions of relationships with others, the objective appraisal of the romantic relationship does not have a bearing on this construct.

Several limitations need to be addressed in the context of these findings. First, this sample was relatively small (*n* = 94) and suffered from significant attrition throughout the daily diary period. Although these rates of attrition are consistent with other intensive longitudinal studies [49], we evaluated and handled the missing data accordingly. Due to this, we accounted for few covariates in the model; future research is encouraged to include additional covariates with larger samples, such as race and ethnicity, sexual orientation, and income. Second, this sample was recruited on the basis of being in a romantic relationship and the presence of SI at baseline. However, despite a high level of SI in the baseline survey, participants reported significantly lower rates of ongoing SI for the remainder of this study. Future research focusing on relational dynamics may need to recruit participants with more severe ideation to determine influences. Further, this study was limited by its examination of relational satisfaction only. Additional relationship measures are encouraged to be included in future studies; for instance, relational conflict, problems, and communication have significant impacts on SI [17] and are likely to have substantial impacts on relational satisfaction as well. Finally, this study only assessed one member of the dyad. While interpersonal factors were examined in the form of relationship satisfaction, thwarted belonging, and perceived burdensomeness, we were unable to include the full dyadic influences on SI, which is recommended in future studies to best understand the complex and bidirectional impacts of each partner on one another [50].

Future research is encouraged to explore alternative analytic plans to further investigate the findings of this study as well as the theoretical application of the ITS to relationship satisfaction and SI. For instance, perceived burdensomeness and thwarted belonging may serve as mediators instead of moderators, as was tested in this study. Theoretically, relationship distress may serve as a risk factor for thwarted belonging and/or perceived burdensomeness, which then serve as risk factors for SI. Although the present study did not find significant associations between relationship satisfaction or thwarted belonging and SI, there is substantial prior evidence for these associations and additional exploration is warranted.

Despite the overall null hypotheses in this study, several clinical implications can be drawn. First, perceived burdensomeness had a significant impact on SI at baseline. The evaluation of what causes a person to feel they are a burden or better off dead are therefore encouraged to be explored, potentially with the insight that the romantic relationship may not be contributing to or exacerbating this experience. Second, though relational satisfaction did not demonstrate an association with SI, relationships remain a consistently listed reason for living [51], prompting clinicians to assess the relationship beyond overall satisfaction.

In summary, this was the first examination of the impacts of romantic relationships on SI in a daily diary study. While romantic relationships have a known influence on individual mental health, including suicide and SI [8], these associations have not been tested using intensive longitudinal methods. The results of this study did not reveal significant associations between relationship satisfaction and SI, even when moderated by thwarted belonging and perceived burdensomeness. Our study highlights areas for future research, such as incorporating additional relational dynamics or measures, investigating those with more severe SI, and including both partners of the dyad.

## Figures and Tables

**Table 1:** Descriptive information of the key variables of adults with suicidal ideation (SI) (*n* = 94).

Variable	*n* Missing	M or %	SD	Range
Age	0	37.47	12.46	20–77
Woman ^a^	0	47.90%	-	-
White ^b^	0	76.00%	-	-
Straight or Heterosexual ^c^	0	61.70%	-	-
Relationship Length	0	9.21	9.87	0–55
Married	0	40.40%	-	0.1
Perceived Burdensomeness	0	18.78	9.65	6–42
Thwarted Belonging	0	34.95	11.32	9–62
Relationship Satisfaction	0	5.20	1.61	1–7
SI Baseline	0	11.30	8.44	1–34
SI Day 1	19	3.69	5.77	0–25
SI Day 2	19	2.67	4.96	0–27
SI Day 3	23	4.05	7.38	0–30
SI Day 4	21	3.91	7.48	0–40
SI Day 5	21	3.01	5.41	0–22
SI Day 6	19	3.85	6.70	0–27
SI Day 7	23	2.41	5.30	0–22
SI Day 8	22	4.31	7.60	0–42
SI Day 9	30	2.97	6.92	0–32
SI Day 10	31	3.09	5.47	0–20

*Notes:* a = reference group is Man or Non-Binary. b = reference group is Alaskan Native/Native American, Asian, Black/African American, Hispanic/Latino, Native Hawaiian/Pacific Islander, and Other. c = reference group is gay, lesbian, bisexual, pansexual, asexual, queer, prefer not to answer, and do not identify as these options.

**Table 2:** Correlations and descriptive information of suicidal ideation, predictors, and covariates in daily diary study (*n* = 94).

	1.	2.	3.	4.	5.	6.	7.	8.	9.	10.	11.	12.	13.	14.	15.	16.
1. D0	-															
2. D1	0.47 *	-														
3. D2	0.39 *	0.74 *	-													
4. D3	0.50 *	0.49 *	0.61 *	-												
5. D4	0.30 *	0.36 *	0.42 *	0.53 *	-											
6. D5	0.32 *	0.42 *	0.48 *	0.42 *	0.51 *	-										
7. D6	0.56 *	0.36 *	0.39 *	0.68 *	0.45 *	0.45 *	-									
8. D7	0.53 *	0.55 *	0.60 *	0.61 *	0.56 *	0.50 *	0.57 *	-								
9. D8	0.41 *	0.25 *	0.29 *	0.26 *	0.65 *	0.43 *	0.29 *	0.31 *	-							
10. D9	0.50 *	0.50 *	0.53 *	0.56 *	0.64 *	0.47 *	0.51 *	0.71 *	0.40 *	-						
11. D10	0.43 *	0.44 *	0.39 *	0.41 *	0.20	0.45 *	0.35 *	0.27 *	0.58 *	0.38 *	-					
12. PB	0.50 *	0.23 *	0.25 *	0.21	0.23 *	0.23 *	0.22	0.30 *	0.25 *	0.41 *	0.31 *	-				
13. TB	0.38 *	0.09	0.16	0.25 *	0.18	0.20	0.20	0.23 *	0.17	0.29 *	0.17	0.53 *	-			
14. RS	0.10	0.01	−0.12	−0.11	−0.14	−0.10	0.11	0.00	−0.03	−0.04	0.09	−0.06	−0.45 *	-		
15. Age	−0.21 *	0.15	0.09	0.06	0.02	−0.05	0.06	−0.05	−0.11	−0.07	−0.12	−0.18	−0.17	−0.16	-	
16. Woman ^a^	−0.10	−0.25 *	−0.23 *	−0.07	−0.17	−0.16	0.04	−0.15	−0.23 *	−0.10	−0.19	−0.07	−0.05	0.01	−0.04	-
*n* Missing	0	19	19	23	21	21	19	23	22	30	31	0	0	0	0	0
M or %	11.30	3.69	2.67	4.05	3.91	3.01	3.85	2.41	4.31	2.97	3.09	18.78	34.95	5.20	37.47	47.90
SD	8.44	5.77	4.96	7.38	7.48	5.41	6.70	5.30	7.60	6.92	5.47	9.65	11.32	8.44	12.46	-
Range	1–34	0–25	0–27	0–30	0–40	0–22	0–27	0–22	0–42	0–32	0–20	6–42	9–62	1–7	20–77	-

*Notes:* a = reference group is Man or Non-Binary. D0 = Baseline. D1 = Day 1. D2 = Day 2, and so forth. * *p* < 0.05.

**Table 3:** Multilevel growth models of suicidal ideation of a 10-day daily diary study (*n* = 94).

	Key Predictors						With Interaction Effects						With Controls					
	Initial Levels			Linear Rates of Changes			Initial Levels			Linear Rates of Changes			Initial Levels			Linear Rates of Changes		
	*b*	*SE*	*p*	*b*	*SE*	*p*	*b*	*SE*	*p*	*b*	*SE*	*p*	*b*	*SE*	*p*	*b*	*SE*	*p*
*Fixed Effects*																		
Estimates	4.44 *	0.47	<0.01	−0.46 *	0.57	<0.01	4.28 *	0.54	<0.01 *	−0.48 *	0.06	<0.01	4.27 *	0.54	<0.01	−0.48 *	0.06	<0.01
RS	0.11	0.34	0.75	−0.05	0.04	0.22	0.26	0.39	0.50	−0.04	0.05	0.44	0.28	0.39	0.47	−0.03	0.04	0.40
PB	0.21 *	0.06	<0.01	0.00	0.01	0.90	0.21 *	0.06	<0.01	0.00	0.01	0.88	0.21 *	0.06	<0.01	−0.00	0.00	0.85
TB	0.02	0.06	0.74	−0.01	0.01	0.07	0.03	0.06	0.64	−0.01	0.01	0.10	0.03	0.06	0.60	−0.01	0.00	0.09
PB*RS							−0.03	0.04	0.47	0.00	0.00	0.61	−0.02	0.03	0.49	−0.00	0.00	0.67
TB*RS							−0.02	0.03	0.64	0.00	0.00	0.70	−0.01	0.03	0.73	−0.00	0.00	0.65
Age													0.02	0.04	0.59	−0.00	0.00	0.74
Woman ^a^													−0.50	0.48	0.31	−0.02	0.05	0.71
*Random Effects*																		
Initial Levels	17.13 *	3.24	<0.01				17.02 *	3.27	<0.01				17.01 *	3.33	<0.01			
Residual	28.24 *	1.50	<0.01				18.31 *	1.50	<0.01				28.41 *	1.51	<0.01			
*Model Fit*																		
Log Likelihood	5133.89						5131.23						5129.45					
# of parameters	10						14						18					
Deviance test							χ^2^ (4) = 2.66, *p* = 0.61						χ^2^ (4) = 1.78, *p* = 0.76					

*Notes:* a = reference group is Man or Non-Binary. RS = Relationship Satisfaction. PB = Perceived Burdensomeness. TB = Thwarted Belonging. All predictors and controls were assessed at baseline. * indicates interaction between variables.

## Data Availability

Due to the sensitive nature of these data, data are not available.
